# Mitigating Clinical Confounding in AI Models: A Comparative Analysis of Associative and Causal-Fused AI for ICU Readmissions

**DOI:** 10.3390/healthcare14142067

**Published:** 2026-07-10

**Authors:** Konstantina Remoundou, Emanuele Koumantakis, Ioanna Roussaki

**Affiliations:** 1 Institute of Communication and Computer Systems, 15773 Athens, Greece; 2 School of Electrical and Computer Engineering, National Technical University of Athens, 15772 Athens, Greece; 3 Center for Biostatistics, Epidemiology, and Public Health, Department of Clinical and Biological Sciences, University of Turin, 10043 Orbassano, Italy; emanuele.koumantakis@unito.it

**Keywords:** ICU readmission, causal discovery, XGBoost, Long Short-Term Memory (LSTM), causal inference, MIMIC-IV

## Abstract

**Introduction**: In critical environments, patient discharge decisions present significant challenges, particularly regarding the prevention of readmissions. However, traditional AI systems focus on correlations rather than causation, resulting in issues related to explainability and generalization. This study conducts a comparative evaluation between standard predictive and causal-fused models to assess their ability to mitigate clinical confounding on predicting readmissions. **Methodology**: Utilizing the MIMIC-IV dataset, we predicted 30-day ICU readmissions through a comparative analysis of associative models (XGBoost, LSTM) and their causal-fused equivalents. The Fast Causal Inference (FCI) algorithm mapped latent confounding via a Partial Ancestral Graph (PAG), while counterfactuals were computed using Causal Forests to estimate the Average Treatment Effect (ATE), which was integrated into the predictive models. Performance was evaluated via AUROC, AUPRC, calibration metrics, precision, recall, and F1-score while feature extraction was used to monitor feature realignment. **Results**: The causal-fused LSTM model maintained a stable AUROC (0.7342 to 0.7357), while the XGBoost AUROC improved (0.6517 to 0.6909). Feature importance extraction revealed a structural realignment; whereas standard models relied heavily on non-actionable frailty proxies such as polypharmacy, causal integration elevated the individualized causal effect of length of stay as a primary predictive driver. The estimated ATE for length of stay was calculated at −0.038. **Conclusions**: The comparison showed that transitioning to causal-fused AI mathematically resolves a clinical Simpson’s Paradox, while also realigning the features based on causal mechanisms without sacrificing predictability. By making this shift, we can mitigate reliance on administrative noise and promote de-confounded interventions rather than passive correlations.

## 1. Introduction

Intensive Care Unit (ICU) readmissions are commonly used to assess the quality of the medical care provided by clinicians, doctors and hospitals. However, even though those events have been vastly researched for the reasons of occurrence and for minimization purposes, unfortunately, they continue to occur. Surprisingly, even in developed countries, hospitals suffer from high ICU readmission rates, given that around 10% of patients will be readmitted back to ICU within a hospital stay [1]. Moreover, there is an escalating trend in the U.S. for ICU readmission rates rising from 4.6% in 1989 to 6.4% in 2003 [2], thus making ICU readmission rates one of the critical quality indicators in the performance evaluation of ICU.

Extending clinical analysis through contemporary epidemiological data, a significant subset of approximately 33% of the abovementioned readmissions seems to occur within the initial 24 h post-discharge, suggesting a critical window of physiological vulnerability [1,2]. The medical impact is most acutely reflected in mortality disparities. While index admission mortality typically remains below 9%, readmitted patients face a profoundly higher in-hospital mortality risk, with rates ranging from 26.8% to 35.6% [2,3]. This “failure to rescue” trajectory is further characterized by an exponential increase in resource utilization, where the total length of hospital stay (LHS) for readmitted cohorts often extends to a mean of 23 to 46 days, effectively doubling or tripling the institutional footprint of a single patient [4,5]. Financially, these events translate into a staggering socioeconomic burden, with costs per readmission frequently exceeding $15,000 to $21,000, often consuming up to 32% of the total index admission budget [6]. Parallel to these quantifiable metrics is the psychological morbidity affecting the medical community and patient families. Readmission triggers a documented rise in Post-Intensive Care Syndrome (PICS), with patient depression rates climbing to nearly 70% post-transfer and 63% of nursing staff reporting burnout linked to the moral distress of managing recurrent, high-acuity clinical failures [7,8]. Consequently, ICU readmission serves as a potent marker of systemic inefficiency, manifesting as a trifecta of clinical deterioration, financial exhaustion, and psychosocial trauma.

Furthermore, the fundamental objective of health, social, and behavioural sciences is predominantly to resolve causal inquiries rather than to simply identify associative patterns. These causal questions, however, necessitate a nuanced understanding of the data-generating process that cannot be derived from raw data and its joint distributions alone. To clarify this distinction, one must recognize that associational concepts, such as correlation, regression, and conditional independence, are defined strictly by the observed distributions of variables. In contrast, causal concepts, which include influence, confounding, and intervention, remain computationally inaccessible from these distributions alone. While standard statistics excel at “controlling for” variables or calculating odds ratios, they lack the structural framework required to manage the confounders, which are the primary barrier when explaining true causal inferences from empirical data [9]. This limitation within traditional statistical models stems from the fact that confounding is an inherently causal phenomenon. While it is the cornerstone of epidemiology, biostatistics, and econometrics, it receives surprisingly little attention in standard statistical texts because it cannot be expressed through purely associational models. Historically, the medical community has bypassed this theoretical bottleneck through the mechanism of Randomized Controlled Trials (RCTs). By physically isolating the treatment effect and nullifying confounding variables, RCTs ensure that the exposed and unexposed groups are exchangeable [10]. In these ideal experimental settings, association effectively becomes causation, allowing researchers to interpret observational measures as direct indicators of therapeutic effect.

However, the clinical reality of observational studies is rarely so straightforward, as the absence of randomization means that exchangeability is not guaranteed. In these complex contexts, association can no longer be interpreted as a proxy for effect, creating an urgent need for a more robust and explicable path to understanding disease etiology. Adopting a causal approach is critical here, as it empowers researchers to address the fundamental “what-if” questions that drive clinical practice, such as the projected survival of a patient if a drug dosage were altered or if an alternative treatment were selected. Distinguishing between the task of outcome prediction and the establishment of a causal relationship is, therefore, not merely a theoretical exercise but a methodological necessity, requiring tools that go beyond simple correlation-based analysis.

To try and mitigate the abovementioned issues, we propose a causal framework for predicting ICU readmissions within a 30-day duration, while in parallel comparing this framework to the state-of-the-art process of prediction through associative AI models. The remainder of this paper is structured as follows: Next section (Section 2) reviews the current state of the art in ICU readmission prediction, comparing the methodological approaches and dataset characteristics of existing studies to identify current literature gaps, closing with the objectives of the study. Section 3 details the methodology, outlining the MIMIC-IV dataset and the applied causal discovery framework used to implement the integration. Section 4 presents the results of the comparative analysis and the predictive evaluation across all models. Section 5 interprets these findings, discussing their clinical impact within the healthcare environment, alongside study limitations and directions for future research. Finally, Section 6 summarizes the findings of the study and the conclusions.

## 2. Related Work

### 2.1. State of the Art

The landscape of predictive modelling for ICU readmissions is characterized by a fundamental branching into two primary methodologies: explainable AI (XAI) frameworks utilizing static clinical datasets and deep learning architectures designed to interpret complex time-series data. While the former prioritizes interpretability through tabular electronic health records (EHR), the latter leverages the high-resolution temporal sequences inherent in intensive care monitoring. To bridge the gap between these approaches, current research increasingly explores ensemble methods to optimize predictive accuracy and model robustness.

For starters, within the domain of interpretable classification, several studies have utilized the Medical Information Mart for Intensive Care (MIMIC-III) database to establish benchmarks. For instance, ref. [11] demonstrated that an XGBoost-based model optimized for Area Under the Receiver Operating Characteristic (AUROC) and Area Under the Precision–Recall Curve (AUPRC) could significantly enhance early detection of readmission risk, surpassing existing state-of-the-art results. Complementing this, ref. [12] evaluated a spectrum of algorithms, including logistic regression, random forest, and gradient descent boosting, to predict “bounce back” risks at multiple clinical time points. Recognizing the challenge of data sparsity often found in EHRs, ref. [13] introduced a weight-decay random forest model. By incorporating a weight-decay term and quantitative eigenvalues to adjust for sparse indicators, their approach improved prediction stability in data-poor scenarios. Following the above, the work of [14] proposed a standardized and explainable machine learning pipeline. Their random forest classification model demonstrated consistent calibration across both monocentric and multicentric settings, providing a scalable framework for diverse clinical environments. Similarly, in a Brazilian university hospital setting, ref. [15] conducted a comprehensive evaluation of eight classification algorithms, spanning Bayesian and ensemble methods. Their analysis of adult patient attributes across three distinct ICUs underscored the importance of feature set selection in achieving high performance across multiple evaluation metrics.

On the other hand, as ICU data is inherently longitudinal, involving time-stamped vital signs and laboratory fluctuations, researchers have shifted toward time-series analysis to capture temporal dependencies. Architectures such as Recurrent Neural Networks (RNNs) and Long Short-Term Memory (LSTM) networks excel at identifying complex patterns within sequential data that traditional static models may overlook. This is exemplified by [16], who utilized the MIMIC-IV database to train RNN, GRU, and LSTM models with integrated attention mechanisms. Their findings confirmed the efficacy of these architectures in predicting not only 30-day readmissions but also in-hospital mortality and prolonged length of stay. Additionally, focusing on specialized populations, ref. [17] developed an LSTM-based model to guide discharge decisions for cardiovascular patients. By benchmarking the LSTM against simpler feedforward networks and logistic regressions, the study highlighted the superior capacity of sequential modelling in handling the complexities of cardiovascular recovery. In a similar approach, ref. [18] employed multivariate LSTM networks to incorporate sudden fluctuations in clinical events, such as glucose levels and heart rate, providing a real-world application of precision medicine by enhancing the accuracy of critical care decision-making. By retaining the same mindset on sequential data, but going beyond pure classification, the integration of survival analysis offers a nuanced perspective on time-to-event data. By considering censored data and time-dependent covariates, models can estimate the probability of readmission within specific windows. The work composed by [19] successfully combined ARIMA time-series modelling with survival analysis, utilizing clinical scores like MODS and NEMS. Their hybrid approach facilitated accurate ICU census forecasting and capacity planning, demonstrating that combining longitudinal trends with survival probability is essential for hospital resource management. Additionally, while focusing on mortality of ICU patients, ref. [20] proposed a robust, data-driven framework, benchmarking three fundamental sequential time-series architectures (FRNN, LSTM and GRU) against a standard feed-forward multilayer perceptron (MLP) baseline. The study achieved high predictive accuracy (AUC of 0.87 to 0.91) across three public databases, while simultaneously providing clear, clinically consistent visual explanations of mortality risk over both variable and temporal dimensions using SHAP.

Finally, to mitigate the risk of overfitting and capture a broader spectrum of predictive patterns, ensemble and stacking methods have emerged as a high-performance alternative. The authors in [21] proposed a two-stage stacking integrated model for cardiovascular patients, where decisions from a diverse first-level set were fused to optimize a second-level classifier. Expanding on this, ref. [22] incorporated clinical severity scoring into a stacked ensemble framework. Their results indicated that integrating severity scores with specialized feature subsets (SetS and SetT) markedly outperformed non-integrated models. Supporting this trend in cardiac care, ref. [23] developed a robust Stacking Ensemble Learner (SEL) designed to detect emergency readmissions in heart disease patients. By effectively identifying pre-attack complications, such ensemble techniques provide a critical safety net, demonstrating the transformative potential of combining model diversity with clinical domain knowledge.

However, AI models come along with some limitations. In the literature review conducted by [24], the authors identified critical systemic bottlenecks that confirm the limitations of current associative approaches by evaluating 24 studies and 49 distinct deep learning models. Those limitations included robustness and trust issues through extreme heterogeneity that was identified across study settings, driven by inconsistent outcome definitions and modelling choices. Furthermore, most of the existing research exhibits a high risk of bias often due to listwise data deletions and improper handling of missing predictors. Following that, the lack of explainability was also an issue in the review as only two studies utilized interpretability tools such as Shapley Additive Explanations (SHAP) to quantify predictor contributions. Even when interpretability is attempted, it remains strictly associated; current models identify peripheral oxygen saturation or heart rate as important features but fail to elucidate the causal pathways driving readmission. Finally, there is profound over-reliance on US-based. This geographic and institutional narrowness leads to “database-specific bias,” resulting in a collapse of performance when models are applied to out-of-distribution populations that concludes to major generalizability issues.

### 2.2. Objective of the Study

Under this light, the overarching research gap identified through this evidence is the complete absence of causal inference in existing ICU readmission prediction strategies. Current models are limited to identifying “what” correlates with a readmission event but cannot address the “why” or simulate the effects of an intervention, while focusing on specific datasets that do not allow the population fairness. Based on that, the objective of this study is to analyze and compare causal-aware AI models to standard associative AI models (hereafter referred to as ‘traditional models’) to demonstrate the potential advancement of the explainability and generalization of the causal-aware models in predicting ICU readmissions. This will hopefully lead to a more robust, etiological and explainable support systems for healthcare professionals in their ICU discharge decisions, especially when they have to handle such a vast amount of data as reported in ICU databases. Specifically, the research aims to:

- Identify and reproduce two state-of-the-art algorithms from existing studies that demonstrate strong performance in ICU readmission prediction and implement them using the MIMIC-IV dataset.
- Enhance those models through causal integration, applying causal discovery and inference techniques to uncover and incorporate underlying cause–effect relationships among clinical variables.
- Compare the performance and interpretability of the causal-fused model with the traditional predictive model to assess adjustments in accuracy, robustness, and clinical explainability.
- Demonstrate how causality-informed AI can move beyond correlation-based predictions to support more reliable, transparent, and actionable clinical decision-making in the ICU setting.

## 3. Materials and Methods

### 3.1. Dataset: MIMIC-IV

The dataset used for this analysis and training is the MIMIC-IV relational database for patients admitted to a tertiary academic medical center in Boston, MA, USA [25]. The dataset is anonymized both in patients’ personal data and in timestamps of crucial events (e.g., admission time, discharge time, death, etc.). All the necessary steps were followed in order to retrieve the dataset based on the guidelines provided by PhysioNet and only the first author of this publication had access to the dataset specifically for this analysis.

The MIMIC-IV dataset consists of data from 364,627 patients from which 65,366 admitted to an ICU, and over 300,000 patients admitted to the emergency department. According to the comparison between hospital and ICU admissions as presented in Table 1, we can identify that more men than women are admitted to the ICU (opposite to hospital admissions, where women have most of the admissions) while the age of overall patients admitted to the ICU is a bit higher on average than those admitted to the hospital by almost 8 years. This is confirmed across genders, although the ages between men and women in both admission types are very close. Additionally, there are no significant discrepancies between the race categories, except for category “other”, which seems to have an increase in ICU admissions. And finally, mortality for those admitted at least once to an ICU is significantly higher, as was expected.

**Figure 1:**
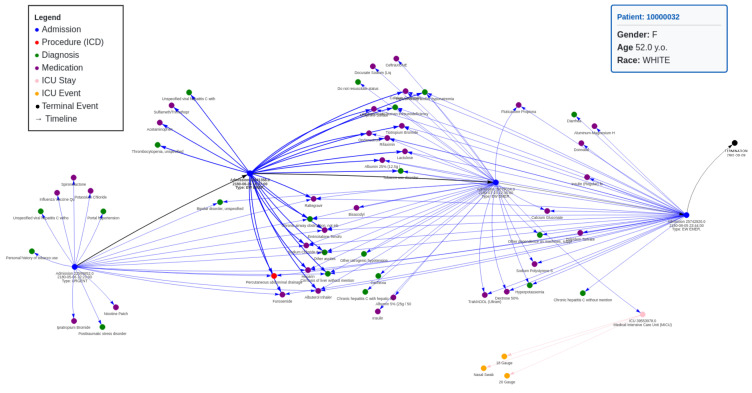
Longitudinal visualization of a patient’s clinical trajectory within the MIMIC-IV dataset. The timeline integrates static demographics, including gender, age, and race, with the occurrence of death and sequential hospital admissions. Each admission details specific disease codes and medications, while the ICU stay highlights granular clinical procedures and temporal dependencies.

However, to understand the complexity and velocity of this dataset, in Figure 1, we can see a clinical trajectory of the patient 10000032 (52-year-old white female) in the dataset, her diseases, the events she underwent and the medications she may have taken until her death. The central temporal axis consists of sequential hospital admissions (blue nodes) linked chronologically, which ultimately culminate in a terminal event (black node). Spreading from each admission node are high-density clusters of specific clinical data points, colour-coded to represent diagnoses (green), medications (purple), and procedures (red). The graph also captures a specific intensive care escalation, depicting an ICU stay (pink node) that branches into granular ICU-specific clinical events (orange nodes). The primary utility of this visualization lies in its explicit representation of clinical complexity and temporal dependencies. The dense accumulation of green and purple nodes at each admission provides a direct visual proxy for the patient’s severe comorbidity burden and polypharmacy.

### 3.2. Data Preprocessing

In this study, an ICU readmission event is defined as an unplanned re-entry into a critical care environment within a 30-day post-discharge window. While narrower definitions such as readmissions occurring strictly within the same hospital admission offer higher clinical specificity, they significantly reduce the available sample size, thereby compromising the training of high-capacity deep learning architectures. This 30-day threshold aligns with most of the contemporary literature, where the majority of similar studies adopt this timeframe to ensure sufficient data volume and standardized benchmarking [24]. The analytical scope further necessitates the management of competing risks, specifically in-hospital mortality. In the proposed framework, death is treated as a competitive risk that invalidates the possibility of readmission. Consequently, patients who succumb during index admission or within the 30-day follow-up period are excluded from the primary cohort. Treating mortality as a non-usable data point ensures that the model’s predictive logic remains focused on the physiological precursors of recurrent critical illness rather than “failure to rescue” signals. Furthermore, in the MIMIC-IV dataset there are a few admissions that do not correspond to the care units that the initial objective is focused on. Some of the abovementioned units are used primarily for pre-surgical preparation or for an intermediate purpose before the patient goes to his room or to the surgery unit. To mitigate the administrative noise and database-specific bias characteristic of large EHR datasets, a readmission is only validated if the patient re-enters one of the pre-specified ICU designated for this study. This ward-specific constraint ensures that the “readmission” reflects a clinical requirement for intensive care rather than a scheduled procedural transfer or an administrative shift between unrelated departments. Furthermore, to facilitate the optimization of deep learning architectures, the final preprocessing stage involved the normalization of continuous variables and the encoding of categorical features to ensure numerical stability and model convergence in addition to a missing value strategy that allowed only patients with no missing values in critical features such as age, gender, race, length of stay and primary disease, while implementing a Bayesian Ridge imputation for the rest.

Finally, a specific strategy of grouping was implemented for the “diseases” variable. In the MIMIC-IV database, diseases are recorded in extreme detail, resulting in thousands of different descriptions for similar conditions. By grouping conditions that share the same underlying pathophysiological mechanism the algorithm is trained to recognize the systemic impact of the specific mechanism on readmission risk, rather than wasting its learning capacity trying to distinguish dozens of clinically equivalent terms. Following that approach, the final categories of diseases were 44 in total. For the medication variable, only the 15 most prevalent types were included, due to computational costs and explainability capacity. Even though there are still many features to be modelled, the known issue of the “curse of dimensionality” is now less prominent.

**Figure 2:**
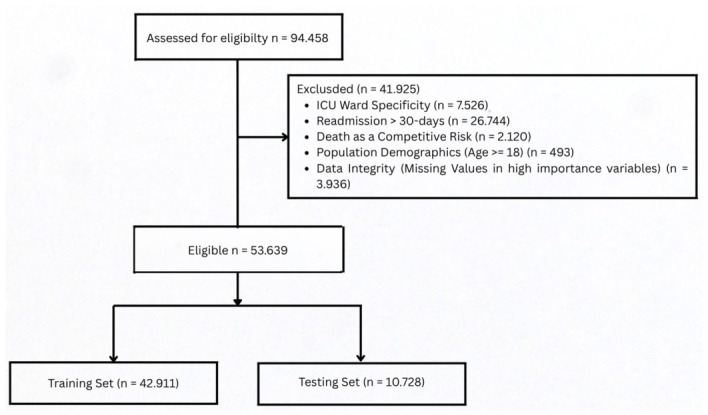
Study flow diagram showing exclusion criteria.

Under this light, the cohort selection and data preprocessing phase followed the inclusion and exclusion criteria shown in Figure 2, to ensure the clinical relevance and statistical integrity. Furthermore, the variables included in the final cohort can be seen in Table 2. This cohort was split in an 80–20 ratio to the training and testing set to be fed in the models.

### 3.3. Causality Integration Approach

The causality framework used for this analysis is the framework that was presented first by Pearl as the Ladder of Causality [26], which serves as a hierarchical map of the cognitive and computational capabilities required to understand causal relationships. It proposes that information is structured into three distinct levels (steps of the ladder), where each higher level necessitates a more complex mathematical framework and a deeper set of assumptions about the underlying data-generating process.

The first level of the ladder is association, which deals with the identification of patterns and regularities in observed data. This level is governed by standard probability and statistics, characterized by the expression P(y|x), the probability of an outcome y given the observation of a condition x. However, this level is fundamentally limited because it cannot distinguish between a variable that causes an outcome and one that is merely a symptom of it. Because associational concepts are defined strictly by the joint distribution of variables, they are “passive”. They describe the world as it is currently observed but remain blind to how the world would change if a new policy or clinical intervention were introduced. This is accomplished through high-dimensional feature extraction using XGBoost and LSTM architectures. These algorithms are proficient at mapping the joint distribution of variables, “seeing” how vital signs or laboratory results coexist with readmission rates.

The second level, intervention, brings the transition from seeing to “doing.” By introducing the do-operator, we simulate the effect of a deliberate action, such as P(y|do(x)). This level is critical for clinical decision support because it allows the model to predict how a patient’s trajectory would change if a specific treatment were actively implemented. Unlike associational models, interventional models require a Directed Acyclic Graph (DAG) or Partial Ancestral Graph (PAG) to serve as an “objective mathematical language” [9]. This level asks “what-if” questions about the future: “If I delay this patient’s discharge by 24 h, how will it affect their risk of readmission?”. Answering such questions is computationally impossible using the raw data from association level alone, because an intervention effectively “breaks” the old rules of the system. For example, if we change the discharge protocol, the old correlations between discharge timing and patient health may no longer hold. To discover this structure from observational EHR data, we implement causal discovery algorithms such as Fast Causal Inference (FCI). Those kinds of algorithms are specifically designed to identify genuine causal influences and uncover fragments of the underlying structure even when some variables remain unobserved, a common occurrence in intensive care workflows. The validity of the applied causal discovery framework relies on three foundational theoretical assumptions that map statistical dependencies to causal structures. First, we assume stability (or faithfulness), positing that the conditional independencies observed within the MIMIC-IV dataset are structural and not the result of coincidental parameter cancellations. Second, the algorithm adheres to causal minimality, ensuring that the resulting graph contains no superfluous connections; every retained edge signifies a mathematically necessary dependency [27]. Finally, because observational data cannot always uniquely determine the direction of every causal relationship [28], the FCI algorithm outputs a Partial Ancestral Graph (PAG). This PAG represents a Markov equivalence class of d-separation, acknowledging that while the algorithm successfully identifies invariant causal skeletons and unconfounded ancestral relationships, certain directional edges remain mathematically indistinguishable without experimental intervention [29].

Finally, the level of counterfactuals, which deals with retrospective reasoning and individual-level hypothetical scenarios, asks what would have happened in the past if we had acted differently. It is characterized by expressions like P(y_x_|e), which represents the probability that an outcome y would have occurred had x been different, given the evidence e of what happened. Processing counterfactuals requires a Structural Causal Model (SCM) that can perform a three-step logical sequence. First, it performs abduction, using the observed evidence to update the state of unobserved patient-specific factors. Second, it performs an action, modifying the model to reflect the hypothetical intervention. Finally, it performs a prediction, computing the new outcome based on the updated model. By reaching this level, AI moves beyond being a mere predictor and becomes a tool for true clinical explanation and individual-level accountability. To quantify these effects and the Average Treatment Effect (ATE), we utilize Causal Forests, a machine learning technique that estimates heterogeneous treatment effects by combining the flexibility of random forests with the structural requirements of causal inference.

The practical integration of this framework follows a formalized empirical workflow through estimation and fusion by utilizing Causal Forests to estimate the magnitude of effects and integrating these results into the initial XGBoost and LSTM models. To extract the most important feature of the model, feature importance was implemented to both models with LSTM integrating the permutation approach [30].

**Figure 3:**
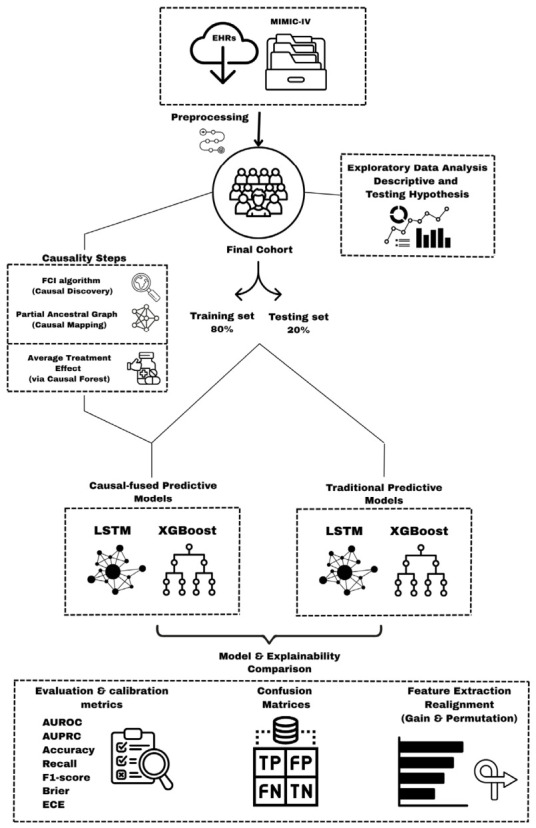
Flow chart showing the methodological workflow of the study.

The methodological approach described above can also be visually seen in Figure 3.

### 3.4. Implementation Details

For the implementation of the above methodology, the Python 3.8.10 environment was utilized, leveraging Sclkit-learn1.3.2 and Tensorflow2.13.1 to parameterize the LSTM and XGBoost architectures. Additionally, for the causal discovery, the FCI algorithm and ATE, econml0.15.1 library was utilized, ensuring the resulting PAG adheres to d-separation equivalence and the principles of minimality and stability. More information about hyper-parametrization and specific utilization can be found here: https://github.com/konrem12/enfield-mimic/tree/main (accessed on 4 May 2026).

All computational experiments, including data preprocessing, causal discovery, and predictive modelling, were executed on a dedicated Linux workstation running Ubuntu 20.04.6 LTS. The core hardware infrastructure was equipped with an AMD Ryzen Threadripper PRO 5945WX 12-Core processor and 64 GB of system RAM and NVIDIA RTX A5000 GPU (24 GB VRAM) leveraging CUDA version 12.9.

## 4. Results

### 4.1. Cohort Demographics and Baseline Characteristics

The final study cohort, derived from the MIMIC-IV dataset and filtered based on the established inclusion criteria, comprised 53,639 adult ICU patients, with 6.162 of them (11.49%) having a readmission within 30 days of their first admission. The following sections detail the demographic, clinical, and modelling results regarding 30-day readmission risk. In Table 3 are shown the general demographic statistics.

Statistical analysis of the age distribution revealed that the readmitted group (n = 6162) was on average older than the non-readmitted group (n = 47,477), with a mean age of 63.14 years compared to 64.92 years. A Welch’s T-test confirmed this difference as statistically significant (T = −7.818, *p* = 6.304 × 10^−15^), a trend further supported by the higher median age (65 vs. 66). Gender representation, on the other hand, showed nearly identical readmission rates between females (11.17%) and males (11.72%), and a Chi-Square Test of Independence (x^2^ = 3.8431, 3 *p* = 0.04995) concluded that gender is a marginally statistically significant predictor of 30-day readmission. Racial background, however, significantly influenced readmission probability (x^2^ = 26.2844, *p* = 2.7729 × 10^−5^). White patients exhibited the highest rate (11.89%), while the Unknown/Other category reported the lowest (10.01%). Additionally, Body Mass Index (BMI) categories demonstrated a highly significant association with readmission status (x^2^ = 21.513, *p* = 6.4778 × 10^−4^), characterized by a non-linear relationship where underweight patients exhibited the highest readmission rate (15.79%) and obese patients the lowest (11.49%).

### 4.2. ICU Ward Specificity and Length of Stay (LOS)

Following the pure demographics analysis, the cohort breakdown by care unit identified the Cardiac Vascular ICU (CVICU) as the largest department (11,201), followed by the Medical ICU (MICU) (10,753), with the Neuro Surgical ICU (Neuro SICU) being the smallest (1058). Despite its volume, the Neuro SICU recorded the highest mean LOS for both non-readmitted (4.01 days) and readmitted patients (7.5 days), whereas the shortest average stays were in the MICU/SICU (2.61 days for non-readmitted and 3.81 days for readmitted patients) and the Coronary Care Unit (CCU) (2.78 days for non-readmitted and 3.11 for readmitted patients). The CCU demonstrated the highest readmission rate at approximately 18%, while the Neuro SICU maintained the lowest at 6%. A Chi-Square Test confirmed a highly significant dependence between ICU Type and readmission probability (*p* = 2.4332 × 10^−55^), and a non-parametric Mann–Whitney U Test established index length of stay (LOS) as a critical predictor (*p* = 1.6241 × 10^−48^), as readmitted patients consistently required longer initial hospitalizations.

### 4.3. Clinical Complexity: Diseases and Medications

Finally, patients presented with a substantial burden of comorbidities, averaging 17.7 diseases (Median = 16) and 42.06 medications (Median = 40) per patient. Primary diagnosis was a profound determinant of risk. Liver-related diseases presented the highest readmission rate (16.77%), followed by Sepsis (15.65%) and pleural-related diseases such as pneumothorax or pleural effusion (15.58%). Other high-risk pathologies included Heart Failure (15.07%), Renal Failure (14.67%), and Spinal Disorder (14.46%). To determine if the specific diagnosis within this high-risk tier significantly dictated the readmission outcome, a Chi-Square Test of Independence was applied to the top 20 diagnoses. The results yielded a Chi2 statistic of 16.33 and a *p*-value of 0.2936, thus showing that there is no statistically significant dependence between the specific disease type and the probability of readmission among these 20 conditions. Detailed readmission rates for diseases are shown on Table 4.

Similarly, complexity was also mirrored in the medication profiles, with a maximum of 196 recordings in a single patient. High-risk medications included Ceftriaxone 1 g/100 mL 0.9% Sodium Chloride (Mini Bag Plus) at 16.67% ratio, and Potassium Chloride 10 mEq ER Tablet at 16.18%. Other highly represented therapeutic regimens in this risk tier included Metronidazole 500 mg/100 mL NS (15.52%), Vancomycin 1000 mg/200 mL Dextrose (Premix) (14.12%), and Piperacillin-Tazobactam 4.5 g/100 mL (13.73%). The vast majority of the cohort (n = 44,547) had no medication from this specific top-tier list recorded, showing a baseline readmission rate of 11.46%. Under the same approach, the test conducted to the top 20 medications showed no statistically significant dependence between the specific disease type and the probability of readmission among these 20 medications. Detailed readmission rates for the medications are shown on Table 5.

### 4.4. Causality Analysis and Predictive Modelling

Causality analysis produced the FCI PAG, illustrating the dependencies between demographic, clinical, and laboratory variables like Blood Urea Nitrogen (BUN) and White Blood Cell count (WBC).

**Figure 4:**
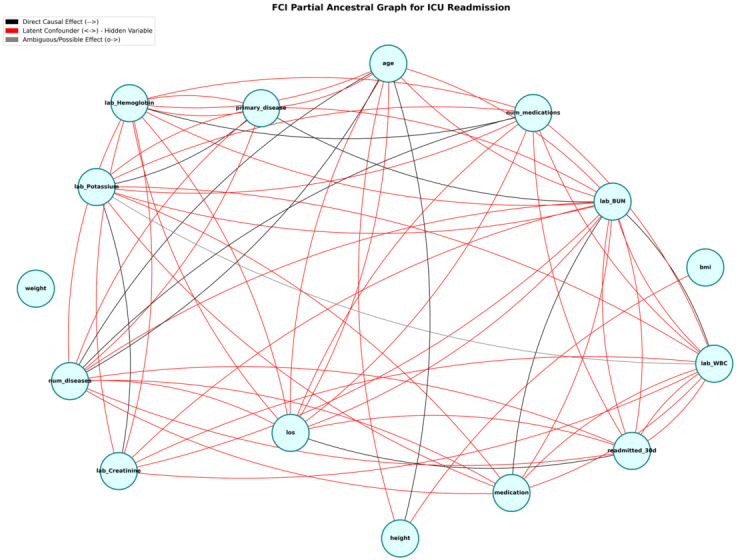
FCI Partial Ancestral Graph for 30-day ICU readmission. This graph illustrates the inferred causal structure and dependencies between demographic, clinical, and laboratory variables within the MIMIC-IV study cohort. The graph highlights the complex interplay of factors, such as age, primary disease, and length of stay, leading to the primary endpoint of 30-day ICU readmission.

In Figure 4, there is a clear indication that most of the variables used in the modelling of the 30-day ICU readmission are actually confounders that have hidden information regarding their relation to the readmission. This can be seen by the numerous red lines of the graph connected to the variable of “readmitted_30d” such as number of diseases and number of medications, as well as, the length of stay, suggesting that there are hidden variables explaining this relationship, not giving a direct cause-and-effect connection.

The performance of the XGBoost and LSTM algorithms, with and without causal regularization, is summarized in Table 2. The integration of the causal meta-feature significantly altered the internal prioritization of the predictive algorithms. Feature extraction through Gain-based importance (XGBoost) and permutation importance (LSTM) revealed that Causal_Effect_LOS became the primary driver of model decisions. The models effectively ‘learned’ to deprioritize high-correlation proxies like polypharmacy in favour of this individualized causal signal. This realignment demonstrates that the counterfactual insight provided more information about the underlying clinical reality than the raw observational variables alone. The ATE score was calculated through Causal Forests at −0.038.

**Figure 5:**
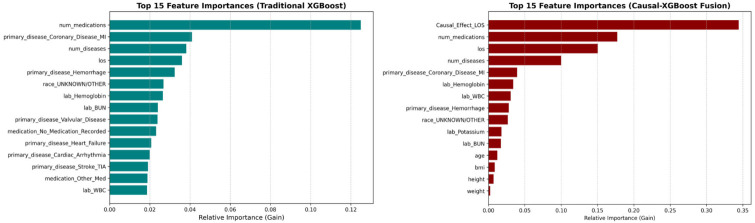
Feature extraction of the top 15 features from the XGBoost algorithm. On the left, the traditional XGBoost algorithm without the causality fusion; on the right, the causality fusioned XGBoost algorithm.

**Figure 6:**
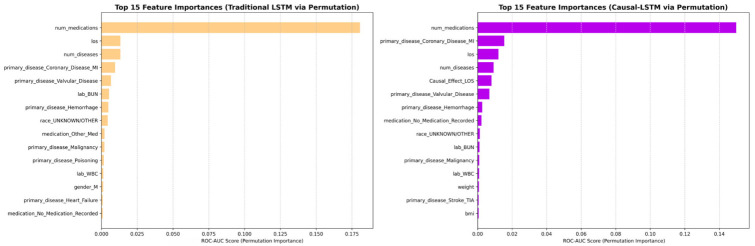
Feature extraction of the top 15 features from LSTM algorithm through permutation importance. On the left, the traditional LSTM algorithm without the causality fusion; on the right, the causality fusioned LSTM algorithm.

Feature importance extraction for the top 15 variables is compared between the traditional and causal-fusion frameworks in Figure 5 and Figure 6.

**Figure 7:**
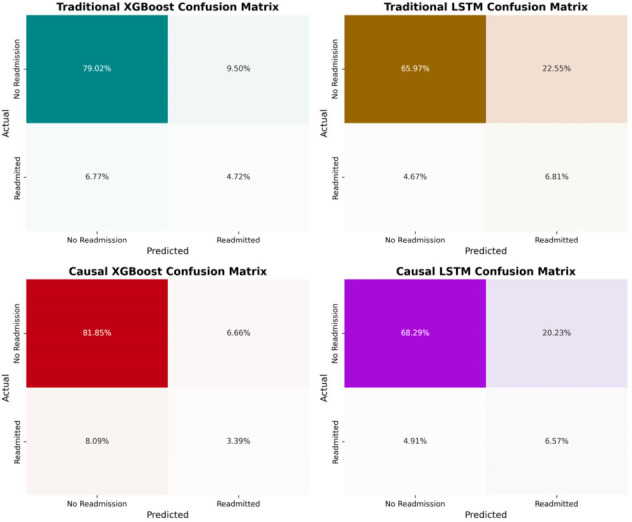
Confusion matrices for all the models. In upper left corner, the traditional XGBoost model confusion matrix; in the upper right corner, the traditional LSTM confusion matrix; in the lower left corner, the causal XGBoost confusion matrix; and in the lower right corner, the causal LSTM confusion matrix.

Finally, the confusion matrices of the four models are shown in Figure 7, below. As seen, the matrices reveal a distinct operational shift induced by causal integration. Specifically, causal fusion systematically reduces the false-positive rate in both architectures—dropping from 9.50% to 6.66% in the XGBoost model and from 22.55% to 20.23% in the LSTM model. However, the identification of actual readmissions (true positives) decreased in the XGBoost model from 4.72% to 3.39%, and marginally in the LSTM model from 6.81% to 6.57%. This is accompanied by a corresponding slight increase in false negatives.

## 5. Discussion

### 5.1. Interpretation of Results and Ethical Implications

Based on the objectives and results, it is obvious that the integration of causal discovery methods into traditional machine learning models does not aim to sharply increase traditional performance metrics. However, evaluating predictive models in clinical environments requires strict adherence to metrics that account for data asymmetry and reliability. Because the patient cohort presents a significant class imbalance, with readmissions constituting roughly 11.5% of the population, numerous metrics were computed to establish that there were no significant changes. Many studies in the literature when evaluating a model only compute AUROC, which is not a sufficient metric according to many researchers, especially when there are rare events occurring, and AUPRC is needed [31,32]. However, there has been a controversy lately in the literature regarding whether AUROC can successfully assess models on imbalanced data [33,34]. In our case, both AUROC and AUPRC reflect this stability across the traditional and causal-fused architectures, showing that forcing the algorithms to potentially change their correlated relationships to causal relationships does not destroy their predictive abilities. From Table 6, it is obvious that the LSTM model consistently provides a better performance baseline than XGBoost, achieving an AUPRC of 0.3255 (traditional) and 0.3280 (causal), compared to the XGBoost baseline AUPRC of 0.2039, which improved to 0.2706 after causal-fusion. On the other hand, probability calibration is equally critical as a model must output reliable risk percentages to foster clinical trust. For the LSTM architecture, causal integration logically refines both the Brier score (improving from 0.1996 to 0.1936) and the ECE (dropping from 0.3183 to 0.3087). Conversely, the causal XGBoost model exhibits a degradation in these metrics, with the Brier score rising to 0.2445 and ECE increasing to 0.3798. This divergence seems expected since the nature of the data is longitudinal and tree-based ensembles may struggle to naturally calibrate synthetic counterfactual variables without aggressive hyperparameter tuning [35,36]. Last, but not least, regarding class-specific metrics, all models demonstrate characteristically low precision for the readmission event (ranging from 0.23 to 0.33) alongside higher recall (up to 0.59 for the traditional LSTM). In intensive care triage, this asymmetry reflects a necessary and mathematically sound trade-off. Minimizing false negatives, namely failing to identify a patient who will deteriorate post-discharge, is prioritized over minimizing false positives, which simply results in prolonged observation [37]. This is also evident by the confusion matrices, where the models’ specificity is enhanced, ensuring that a readmission prediction is driven by a verified mechanistic signal rather than an opportunistic correlation. The causal-fusion models maintain this necessary clinical sensitivity, prioritizing a balanced F1-score (0.31 to 0.34) for the event class without reducing the recall required for patient safety.

The most fundamental finding of this framework is the radical realignment of feature prioritization. In the traditional XGBoost model, the total number of medications (num_medications) dominates as the strongest risk predictor. While statistically valid as a proxy for polypharmacy and clinical frailty, this factor is not inherently actionable; clinicians cannot arbitrarily discontinue necessary treatments to reduce readmission probability. This is also confirmed by the FCI PAG which maps the structural limitations of the observational data, proving visually and mathematically that most clinical variables recorded in the electronic health records do not have a direct, isolated cause-and-effect relationship with the readmission event. As illustrated in the graphical output, the dense network of red lines connecting variables to the “readmitted_30d” endpoint confirms a high degree of unobserved confounding. The PAG in Figure 3 explicitly exposes the number of medications as a confounded proxy, linked via red lines to the outcome, which indicates that hidden variables are driving the relationship. Under this light, while statistically valid as a marker for polypharmacy and clinical frailty, this factor is not inherently actionable; clinicians cannot arbitrarily discontinue necessary treatments to reduce readmission probability. Conversely, the integration of the causal meta-feature realigns this prioritization, highlighting the causal effect of the length of stay (Causal_Effect_LOS) as the primary driver of the algorithm’s decisions. This translates into a quantified clinical rule through the ATE, calculated via Causal Forests at −0.038. In hospitals, this indicates that prolonging the length of stay for frail patients has a causal tendency to reduce the risk of 30-day readmission by 3.8%. The system mathematically resolves a clinical Simpson’s Paradox, proving that extending hospitalization actively reduces readmission risk and demonstrating that discharge timing can serve as a safe, adjustable variable for targeted intervention [38,39,40,41].

Following the initial evaluation metrics and causality discovery, the algorithmic comparison of the two models before and after causal fusion via feature importance extraction showed that, for XGBoost, the integration of causal meta-features drastically realigned the decision trees by providing a single, high-information signal. Rather than experiencing a standard “predictive-causal tradeoff” as it is expected from causal predictive models [42,43], the XGBoost model demonstrated a distinct increase in AUROC, rising from 0.6517 to 0.6909. This confirms that elevating Causal_Effect_LOS to the absolute primary predictive driver (replacing num_medications) actively resolved confounding noise that had previously hindered the associative model. On the other hand, the causal LSTM retained num_medications as its primary feature, while successfully embedding Causal_Effect_LOS into the top five predictive drivers. This discrepancy is logically sound when considering the architecture of deep neural networks and the permutation methodology, by penalizing the disruption of correlated, densely interconnected variables (like a patient’s overall medication load) [44,45]. Yet, the successful embedding of the counterfactual signal (Causal_Effect_LOS) high in the hierarchy proves that the network did not ignore the causal logic; rather, it fused the baseline frailty marker with the interventional lever.

Based on the above, transitioning to causal-fused architectures can help address persistent ethical challenges in healthcare AI. Traditional deep learning models, such as the baseline LSTM, are often criticized as “black boxes” because they struggle to explain the reasoning behind their predictions. The causal-fusion approach improves interpretability by embedding a mathematically defined clinical mechanism (the causal LOS) directly into the model’s decision process. While this does not make the entire network perfectly transparent, it links a major part of the algorithm’s logic to a real-world intervention rather than opaque correlations. This shift may also improve model generalizability. Standard associative algorithms frequently fail when deployed in new clinical settings because they overfit the specific administrative data of their training environment, leading to severe “database-specific bias” [24,41]. By using the FCI PAG to map latent confounding and prioritizing causal mechanisms, the framework focuses on relationships that are naturally more stable across different patient populations. Although multicenter validation is still required, this method systematically reduces the model’s reliance on site-specific noise. Finally, integrating causal discovery may act as a practical safeguard for data fairness. Observational health records often contain historical biases, and standard associative models risk absorbing and amplifying these disparities. By evaluating hypothetical interventions, specifically, by isolating the ATE of extending an ICU stay, the framework mathematically separates the true interventional impact from underlying demographic confounders [9]. Consequently, causal-integrated AI represents a structured step toward more equitable healthcare tools, prioritizing objective treatments over historically biassed data structures.

### 5.2. Limitations

While this study introduces a robust methodological framework for causal-predictive fusion in ICU readmissions, there are a few crucial limitations that must be acknowledged, primarily related to the nature of the data and the inherent constraints of causal discovery algorithms.

First, the analysis relies exclusively on observational data from a single centre and under this light, the external validity of the causal-fused LSTM model remains to be established in varied healthcare systems with different discharge protocols and demographic profiles. Furthermore, despite the application of advanced causal inference techniques (such as Causal Forests and the FCI algorithm), the study is inherently observational and lacks experimental validation. Thus, the causal estimates, including the counterfactual effect of the length of stay (LOS), should be interpreted as strong, data-driven hypotheses rather than definitive clinical directives.

Second, the computational complexity of the MIMIC-IV dataset necessitated a strategic reduction of the feature space. We focused on a clinically curated subset of core variables (e.g., demographics, length of stay, and essential laboratory markers). To manage dimensionality in the AI algorithms used (XGBoost and LSTM) without losing the broader clinical picture, complex medical histories were condensed into quantitative proxies, specifically the comorbidity burden (num_diseases) and polypharmacy count (num_medications). While these proxies proved to be exceptionally strong predictors of patient frailty, they inevitably result in a loss of granular, qualitative information (e.g., the specific pharmacological interactions or the severity of individual comorbidities). Following this limitation, we are obligated to mention that this reduction in granularity leaves the models susceptible to residual, unmeasured confounding. Critical variables that heavily influence readmission risk, such as a patient’s socioeconomic status, adherence to medication post-discharge, access to outpatient care, or other non-structural information such as images, notes, etc., are absent from the dataset.

Finally, the temporal dynamics of the patients’ physiological states were simplified. Although the LSTM architecture is designed to handle sequential data, the current experimental setup utilized more of a static approach to this trajectory. For example, it utilized the last recorded laboratory values prior to discharge only without taking into account the overall trajectory of the patient’s admission. This approach captures the patient’s state at a critical decision-making time but does not account for the continuous physiological trajectories (e.g., the rate of improvement or deterioration during the ICU stay), which could provide deeper mechanistic insights into readmission risk.

### 5.3. Future Work

Future research should focus on integrating the abovementioned limitations while also addressing other critical advancements. More specifically, to overcome the single-centre constraints of the MIMIC-IV database, subsequent studies must prioritize the external validation of the proposed hybrid models. Testing the transferability of the causal LSTM and causal XGBoost frameworks on diverse, multicenter datasets will be essential. This step is critical to assess algorithmic fairness and ensure that the causal estimates and predictive performance generalize reliably across different hospital systems, discharge protocols, and diverse patient demographics. Additionally, future iterations must utilize a significantly larger subset of clinical variables to provide a more comprehensive view of patient frailty and mitigate unmeasured confounding [34]. While our current study was constrained by the computational memory limits inherent from when handling big data, leveraging more scalable causal network structures will allow for the inclusion of broader feature sets. Integrating high-dimensional data, such as social determinants of health and unstructured clinical notes, will offer a more complete identification of the confounders driving regional and systemic health disparities [46].

Moving toward approach advancements, transitioning from a static “discharge snapshot” to a dynamic, time-aware architecture is a critical next step. Future research should integrate temporal variables to capture the continuous physiological effect of time during the ICU stay. By modelling the longitudinal trajectories of vital signs, laboratory trends, and sequential treatment responses, rather than relying solely on their final values prior to discharge, causal models can better identify the dynamic rates of patient deterioration or recovery [35]. Finally, the analytical pipeline should be expanded by training and testing a wider array of advanced machine learning and deep learning algorithms. Beyond standard recurrent architectures and tree-based ensembles, future work should explore the integration of Time-Series Transformers and Causal Graph Neural Networks (GNNs) [47]. Causal GNNs offer a highly promising path for directly embedding the causal relationships of complex, multi-modal biomedical data into the neural network architecture itself, enabling the discovery of invariant mechanistic pathways rather than relying on spurious correlations.

## 6. Conclusions

Intensive Care Unit (ICU) readmissions are a critical quality metric. However, standard predictive machine learning models often rely on confounded associative proxies rather than actionable mechanisms. To evaluate the methodological shift from correlation to causation, this study conducted a comparative analysis between standard associative artificial intelligence and causal-fused architectures. Using the MIMIC-IV dataset, latent confounding was mapped through the Fast Causal Inference (FCI) algorithm, while counterfactuals computed via Causal Forests were integrated into XGBoost and LSTM algorithms.

The technical comparison demonstrates that causal integration fundamentally realigns algorithmic decision-making logic while strictly preserving, and in some cases enhancing, discriminative power. The causal-fused LSTM maintained a robust baseline AUROC of 0.7357, while the causal-fused XGBoost demonstrated a clear improvement, increasing its AUROC from 0.6517 to 0.6909 and reducing its false-positive rate from 9.50% to 6.66%. The methodological novelty lies in the structural feature realignment: while traditional models prioritized non-actionable statistical proxies like polypharmacy, the causal architectures successfully elevated the individualized impact of discharge timing as the primary predictive driver. Supported by an extracted Average Treatment Effect of −0.038, this framework mathematically resolves a clinical Simpson’s Paradox, providing quantified evidence that extending hospitalization for frail patients actively reduces 30-day readmission risk by 3.8%. Ultimately, the above provides strong supporting evidence that transitioning to causal-fused AI mitigates reliance on administrative noise, empowering clinicians to base discharge protocols on transparent, de-confounded interventions rather than passive correlations.

## Figures and Tables

**Table 1:** Comparison of MIMIC-IV demographics, between hospital and ICU patients.

		Patients Admitted to Hospital N (%) or Mean (sd)	Patients Admitted to ICU N (%) or Mean (sd)
Gender	Female	117,736 (52.69)	28,646 (43.82)
	Male	105,716 (47.31)	36,720 (56.18)
Age (mean)	Overall	55.62 (20.24)	63.41 (17.07)
	Female	54.77 (21.24)	64.53 (17.78)
	Male	56.58 (19.00)	62.54 (16.45)
Race	WHITE	146,885 (65.73)	43,247 (66.16)
	BLACK	28,366 (12.69)	6033 (9.23)
	UNKNOWN/OTHER	26,319 (11.78)	11,737 (17.96)
	HISPANIC/LATINO	12,321 (5.51)	2361 (3.61)
	ASIAN	9561 (4.28)	1988 (3.04)
Mortality	Overall	36,882 (16.51)	21,942 (33.57)
	Female	17,465 (14.84)	9979 (29.04)
	Male	19,417 (18.36)	11,963 (38.59)

**Table 2:** Variables included in the final cohort based on the inclusion/exclusion criteria of the ICU readmission case.

Variable	Type	Description	Use in the Experiment
subject_id	Integer/Categorical	A unique identifier assigned to a single patient. It remains the same across all their hospital visits.	Identification
hadm_id	Integer/Categorical	A unique identifier for a specific hospital admission. A subject_id can have multiple hadm_ids.	Identification
stay_id	Integer/Categorical	A unique identifier for a specific ICU stay. A single hospital admission (hadm_id) can have multiple ICU stays. (Used only for identification purposes, not modelling)	Identification
first_careunit	Categorical (String)	The specific type of ICU the patient was first admitted to (e.g., MICU, SICU, CCU).	Feature Derivation
last_careunit	Categorical (String)	The specific type of ICU the patient was discharged from.	Feature Derivation
intime	Datetime	The exact date and time the patient was transferred to the ICU.	Feature Derivation
outtime	Datetime	The exact date and time the patient was transferred out of the ICU.	Feature Derivation
los	Numeric (Float)	The number of days that the patient stayd in the ICU computed from „intime” and „outtime”	Modelling
race	Categorical (String)	The patient’s self-reported race or ethnicity.	Modelling
gender	Categorical (String)	The patient’s gender (usually “M” or “F”).	Modelling
age	Numeric (Float/Int)	The actual calculated age of the patient at the time of this specific admission.	Modelling
height	Numeric (Float)	The patient’s height (typically recorded in inches or centimetres).	Modelling
weight	Numeric (Float)	The patient’s weight (typically recorded in pounds or kilograms).	Modelling
bmi	Numeric (Float)	Body Mass Index, calculated from height and weight.	Modelling
primary_disease	Categorical (String)	The main diagnostic category or reason for the patient’s hospital admission (e.g., “Sepsis”, “Heart_Failure”).	Modelling
medication	Categorical (String)	The primary medication of interest prescribed to the patient, or a grouped category of their primary treatment.	Modelling
num_diseases	Numeric (Integer)	The total count of comorbid conditions/diagnoses the patient has (Comorbidity Burden).	Modelling
num_medications	Numeric (Integer)	The total count of distinct medications prescribed to the patient at discharge (Polypharmacy proxy).	Modelling
lab_Creatinine	Numeric (Float)	Indicator of kidney function. High levels suggest acute or chronic kidney injury.	Modelling
lab_Potassium	Numeric (Float)	An essential electrolyte. Abnormal levels pose severe risks for cardiac arrhythmias.	Modelling
lab_BUN	Numeric (Float)	Blood Urea Nitrogen; an indicator of kidney function and hydration status.	Modelling
lab_Hemoglobin	Numeric (Float)	Indicates the blood’s oxygen-carrying capacity. Low levels represent anemia (bleeding/frailty).	Modelling
lab_WBC	Numeric (Float)	White Blood Cell count. Elevated levels typically indicate an active infection or severe inflammation.	Modelling

**Table 3:** Demographic statistics of the final cohort.

	Mean	std	25%	50%	75%
age	63.34	17.72	53.00	65.00	76.00
bmi	36.46	710.96	24.00	27.50	32.01
height	168.77	143.72	160.02	167.64	175.26
weight	131.95	8405.60	66.50	79.38	94.85
num_diseases	17.70	9.26	11.00	16.00	23.00
num_medications	42.07	19.97	27.00	40.00	54.00
los_days	3.19	4.82	1.08	1.83	3.25
lab_Creatinine	1.14	1.15	0.70	0.80	1.10
lab_Potassium	4.10	0.46	3.80	4.10	4.40
lab_BUN	21.20	16.37	11.00	17.00	25.00
lab_Hemoglobin	10.42	1.94	8.90	10.20	11.70
lab_WBC	8.92	5.55	6.30	8.20	10.50

**Table 4:** Top 20 Diseases reported as a primary disease of the ICU patients sorted in response to readmission rate.

Primary_Disease	Total ICU Patients	Readmissions	Readmission Rate (%)
Liver_Disease	632	106	16.77
Sepsis	1521	238	15.65
Pleural_Disease	199	31	15.58
Heart_Failure	1818	274	15.07
Renal_Failure	184	27	14.67
Spinal_Disorder	325	47	14.46
IBD_Colitis	104	15	14.42
Hematologic_Vascular_Disease	277	39	14.08
Malignancy	2369	331	13.97
Endocrine_Disorder	81	11	13.58
GI_Symptom_Condition	85	11	12.94
Substance_Tox	891	115	12.91
Respiratory_Failure	863	110	12.75
Medical_Device_Surgical_Complication	780	99	12.69
Hemorrhage	2291	290	12.66
Neuropathy_Nerve_Disorder	99	12	12.12
Aortic_Aneurysm_Dissection	1140	137	12.02
Valvular_Disease	4099	481	11.73
GI_Structural_Disorder	447	52	11.63
Other_Infection	768	89	11.59

**Table 5:** Top 20 medications provided to the patients sorted in response to readmission rate.

Medication	Total ICU Patients	Readmissions	Readmission Rate (%)
CeftriaXONE 1 g/100 mL 0.9% Sodium Chloride (Mini Bag Plus)	54	9	16.67
Pantoprazole 40 mg Via	111	18	16.22
Potassium Chloride 10 mEq ER Tablet	68	11	16.18
Metronidazole 500 mg/100 mL NS	58	9	15.52
Vancomycin 1000 mg/200 mL Dextrose (Premix)	85	12	14.12
Piperacillin-Tazobactam 4.5 g/100 mL Iso-Osmotic Dextrose	51	7	13.73
Heparin_ 25,000 Units/250 mL 5% DextrosePremix Bag	236	32	13.56
Acetaminophen 500 mg Tablet	324	43	13.27
CefePIME 2 g Vial	54	7	12.96
Sodium Chloride 0.9% Flush	1033	128	12.39
Aspirin (Chewable) 81 mg Tab	57	7	12.28
Docusate Sodium 100 mg Capsule	174	20	11.49
No_Medication_Recorded	44,547	5104	11.46
OxyCODONE (Immediate Release) 5 mg Tablet	133	14	10.53
Senna 8.6 mg Tablet	67	7	10.45
Insulin Human Regular 100 Units/100 mL 0.9% Sodium Chloride	81	8	9.88
CefTRIAXone 1 g Vial	51	5	9.80
Heparin Sodium 5000 Units/mL- 1 mL Vial	221	21	9.50
Acetaminophen 325 mg Tablet	169	16	9.47
Sodium Chloride 0.9%-Floor Stock Bag	419	38	9.07

**Table 6:** Results of metrics (AUROC, Precision, Recall, F1-score) of XGBoost and LSTM prediction algorithms on the ICU readmission prediction for the patients in cohort.

		Traditional Models		Causal-Fused Models	
		XGBoost	LSTM	XGBoost	LSTM
	AUROC	0.6517	0.7342	0.6909	0.7357
	AUPRC	0.2039	0.3255	0.2706	0.328
	Brier Score	0.1947	0.1996	0.2445	0.1936
	ECE	0.3108	0.3183	0.3798	0.3087
Precision	No Readmission	0.92	0.93	0.92	0.93
	Readmission	0.33	0.23	0.23	0.25
Recall	No Event	0.89	0.75	0.80	0.77
	Event	0.41	0.59	0.47	0.55
F1-score	No Event	0.91	0.83	0.85	0.84
	Event	0.37	0.33	0.31	0.34

## Data Availability

Data and information of data for this study can be obtained in https://physionet.org/content/mimiciv/3.1/ (accessed on 15 December 2024).

## Abbreviations

The following abbreviations are used in this manuscript:

ARIMA	Autoregressive Integrated Moving Average
ATE	Average Treatment Effect
AUPRC	Area Under the Precision-Recall Curve
AUROC	Area Under the Curve
BMI	Body Mass Index
BUN	Blood Urea Nitrogen
DAG	Directed Acyclic Graph
ECE	Expected Calibration Error
EHR	Electronic Health Records
FCI	Fast Causal Inference
GRU	Gated Recurrent Unit
ICU	Intensive Care Unit
LHS	Length of Hospital stays
LOS	Length of Stay
LSTM	Long Short-Term Memory
MIMIC	Medical Information Mart for Intensive Care
MODS	Multiple Organ Dysfunction Score
NEMS	Nine Equivalents of Nursing Manpower Use Score
PAG	Partial Ancestral Graph
PICS	Post-Intensive Care Syndrome
RCTs	Randomized Clinical Trials
RNN	Recurrent Neural Nerworks
SCM	Structural Causal Model
SEL	Stacking Ensemble Learner
SHAP	SHapley Additive exPlanations
WBC	White Blood Cell count
XAI	Explainable AI
